# Progress in soybean functional genomics over the past decade

**DOI:** 10.1111/pbi.13682

**Published:** 2021-08-25

**Authors:** Min Zhang, Shulin Liu, Zhao Wang, Yaqin Yuan, Zhifang Zhang, Qianjin Liang, Xia Yang, Zongbiao Duan, Yucheng Liu, Fanjiang Kong, Baohui Liu, Bo Ren, Zhixi Tian

**Affiliations:** ^1^ State Key Laboratory of Plant Cell and Chromosome Engineering Institute of Genetics and Developmental Biology Innovative Academy for Seed Design Chinese Academy of Sciences Beijing China; ^2^ Innovative Center of Molecular Genetics and Evolution School of Life Sciences Guangzhou University Guangzhou China; ^3^ State Key Laboratory of Plant Genomics Institute of Genetics and Developmental Biology Innovative Academy for Seed Design Chinese Academy of Sciences Beijing China; ^4^ University of Chinese Academy of Sciences Beijing China

**Keywords:** soybean, functional genomics, omics, yield components, seed composition, stress resistance, nodulation, domestication, transgenic technology

## Abstract

Soybean is one of the most important oilseed and fodder crops. Benefiting from the efforts of soybean breeders and the development of breeding technology, large number of germplasm has been generated over the last 100 years. Nevertheless, soybean breeding needs to be accelerated to meet the needs of a growing world population, to promote sustainable agriculture and to address future environmental changes. The acceleration is highly reliant on the discoveries in gene functional studies. The release of the reference soybean genome in 2010 has significantly facilitated the advance in soybean functional genomics. Here, we review the research progress in soybean omics (genomics, transcriptomics, epigenomics and proteomics), germplasm development (germplasm resources and databases), gene discovery (genes that are responsible for important soybean traits including yield, flowering and maturity, seed quality, stress resistance, nodulation and domestication) and transformation technology during the past decade. At the end, we also briefly discuss current challenges and future directions.

## Introduction

Cultivated soybean (*Glycine max* [L.] Merr.) was domesticated from wild soybean (*G*. *soja* Sieb. & Zucc.) in China approximately 5000 years ago, after which was spread worldwide (Carter *et al*., 2004; Wilson, 2008). Currently, soybean has become one of the most economically important oilseed and biodiesel crops and also serves as a main source of protein and oil for both human food and animal feed (Hartman *et al*., 2011). Early soybean breeding mainly relied on repeated selection of preferred seeds by farmers from cultivated population. Starting from the early 1900s, artificial hybridization was applied. The first modern soybean cultivar developed by hybridization was released in North American breeding programmes in 1940s (Rincker *et al*., 2014; Wolfgang and Charles, 2017). Afterwards, artificial hybridization started to be widely incorporated in soybean breeding (Anderson *et al*., 2019; Li *et al*., 2001). Artificial hybridization greatly expanded the genetic base of developed lines and significantly improved soybean adaptation and production (Anderson *et al*., 2019).

With the arising and developing of molecular biology technology, marker‐assisted selection (MAS) was used to speed up the breeding process, particularly in development of disease‐ and insect pest‐resistant cultivars (Li *et al*., 2020b). Using different kinds of genetic markers, linkage and physical maps were constructed (Chan *et al*., 2012; Cregan *et al*., 1999; Marek *et al*., 2001; Song *et al*., 2004). By integrating available genetic maps and physical maps, the Consensus Map 4.0 was built (Hyten *et al*., 2010a; Hyten *et al*., 2010b). Using these markers, large numbers of quantitative trait loci (QTLs) affecting related traits have been identified in soybean. However, the limited number of molecular markers and their uneven distribution limited the efficiency and accuracy of QTL positioning.

**Figure 1:**
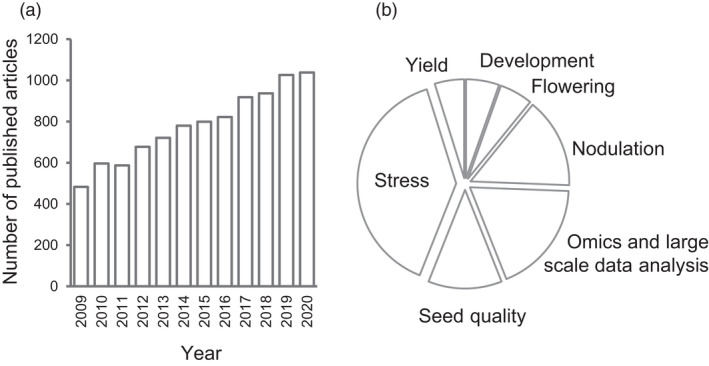
Statistics of publications with term ‘soybean’ from 2009 to 2020. (a) The number of publications with the term ‘soybean’ in each year. (b) The percentage of publications with the term ‘soybean’ and the indicated terms in different research fields.

The reference genome of a cultivated accession (Williams 82) was released in 2010 (Schmutz *et al*., 2010), which brought about the era of soybean functional genomics (Chan *et al*., 2012; Li *et al*., 2017a; Wang and Tian, 2015; Xia *et al*., 2013). Benefit of having the reference genome, research publications on soybean have almost doubled compared with ten years ago, with higher ratios on stress, omics and nodulation (Figure 1). Here, we review the advances in soybean functional genomics and transformation technology during the past decade and discuss the challenges and prospects for future soybean functional genomic studies.

## Progress in omics

Advances in sequencing technologies, particularly in long‐read sequencing, have led to the production of improved assembly genomes (Burton *et al*., 2013; Yang and Huang, 2018). After the release of the reference genome based on Williams 82, Kim *et al*. (2010) assembled a genome sequence of a wild soybean (*G. soja* var. IT182932) using Illumina‐GA and GS‐FLX. Shen *et al*. *de novo* assembled a high‐quality genome for cultivar ‘Zhonghuang 13’ (Gmax_ZH13) using single‐molecule real‐time (SMRT) sequencing, optical mapping, chromosome conformation capture sequencing (Hi‐C) and next‐generation sequencing (HiSeq) (Shen *et al*., 2019; Shen *et al*., 2018a). Xie *et al*. (2019) then assembled a high‐quality reference genome for wild soybean W05 in 2019. Valliyodan *et al*. (2019) *de novo* assembled references for another two cultivars and one wild soybean using a combination of short‐ and long‐read technologies. In addition to the *de novo* assembly of reference genomes, pan‐genome also progressed significantly in soybean. In 2014, Li *et al*. reported the first soybean pan‐genome built by assembly of seven wild soybeans decoded using second‐generation sequencing technology (Li *et al*., 2014b). Recently, Liu *et al*. (2020f) constructed a soybean pan‐genome by *de novo* assembly of 26 representative wild and cultivated soybeans using long‐read sequencing. This assembly produced not only golden‐grade genomes for each accession, but also for the first time reported a graph‐based genome in plants, which provides a promising platform for future in‐depth soybean functional genomic studies (Liu and Tian, 2020; Lyu, 2020; Tao *et al*., 2020; Willson, 2020).

The reference genome sequences revealed that soybean had undergone at least two whole‐genome duplication (WGD) events within the last 60 million years (Schmutz *et al*., 2010). It was found that approximately 50% of the paralogs arising from the WGD had undergone subfunctionalization at the expression level in soybean, suggesting that the main consequence of WGD in soybean may be at the regulatory level (Roulin *et al*., 2013). The diversification of WGD genes was confirmed by the evolutionary and functional analyses several gene families (Li *et al*., 2016b; Wang *et al*., 2015d; Yang *et al*., 2016; Zhang *et al*., 2017). It was also found that, despite the WGD, the genes in networks with a high level of connectivity were more conserved than those with low connectivity (Kim *et al*., 2015; Severin *et al*., 2011). Moreover, WGD genes exhibited a decreased frequency of alternative splicing compared with non‐WGD genes, which was associated with their reduced intron length, exon number and transcriptional level (Shen *et al*., 2014). WGD also affected the evolution of transposable elements (Du *et al*., 2010b; Du *et al*., 2012; Tian *et al*., 2012).

Soybean population genetics has also greatly advanced in the past ten years due to the availability of a soybean reference genome and the development of next‐generation sequencing technology. The resequencing of dozens of wild and cultivated soybean accessions revealed the consequences of the artificial selection accompanying domestication and showed that genetic diversity was significantly decreased after domestication (Lam *et al*., 2010; Li *et al*., 2013b). Integration of selection sweep and genome‐wide association studies (GWAS) on domestication traits using the data from a resequencing of 302 wild soybeans, landraces and improved cultivars disclosed the genetic loci responsible for traits related to soybean domestication, improvement and local breeding (Zhou *et al*., 2015). Moreover, it was found that naturally occurring introgression was widespread and counteracted genetic bottlenecks during soybean domestication (Wang *et al*., 2019e). Hundreds of small RNAs had been identified in soybean (Arikit *et al*., 2014; Golicz *et al*., 2018; Lin *et al*., 2020a; Wong *et al*., 2011; Zhou *et al*., 2013), some of which showed tissue‐specific or time‐specific transcriptional patterns, indicating their biological relevance (Arikit *et al*., 2014; Lin *et al*., 2020a). Population genetic analyses suggested a co‐evolution of *MIRNA* and miRNA targets during soybean domestication (Liu *et al*., 2016a; Zhao *et al*., 2015a).

So far, over 6000 soybean accessions have been genotyped to investigate the footprints of breeding (Chung *et al*., 2014; Fang *et al*., 2017; Han *et al*., 2016; Kajiya‐Kanegae *et al*., 2021; Liu *et al*., 2020f; Qi *et al*., 2021; Qiu *et al*., 2014; dos Santos *et al*., 2016; Shimomura *et al*., 2015; Torkamaneh *et al*., 2018; Valliyodan *et al*., 2016; Zhou *et al*., 2015) (Table 1). The large amount of resequencing data has generated a great number of nucleotide polymorphism (SNP) markers, which greatly facilitate the haplotype map construction (Torkamaneh *et al*., 2021) and SNP Arrays development (Lee *et al*., 2015; Song *et al*., 2020; Wang *et al*., 2016a), and also increased the efficiency and accuracy of gene/QTL mapping (Baird *et al*., 2008; Bandillo *et al*., 2017; Lee *et al*., 2015; Patil *et al*., 2016).

**Table 1:** Whole‐genome sequencing in soybean.

Accession information	Method	Accession number	Reference
Williams 82 (cultivar)	De novo sequencing and assembly	GCA_000004515.3	Schmutz *et al*. (2010)
14 cultivars; 17 wilds	Re‐sequencing	SRA020131	Lam *et al*. (2010)
T182932 (wild)	De novo sequencing and assembly	SRA009252	Kim *et al*. (2010)
10 cultivars; 5 wilds	Re‐sequencing	ERP002622	Chung *et al*. (2014)
7 wilds	De novo sequencing and assembly	PRJNA195632	Li *et al*. (2014b)
9 semi‐wilds; Maliaodou (semi‐wild); Lanxi1(wild)	Re‐sequencing; De novo sequencing and assembly	PRJNA227063	Qiu *et al*. (2014)
W05 (wild)	De novo sequencing and assembly	GCA_000722935.2	Qi *et al*. (2014)
240 cultivars; 62 wilds	Re‐sequencing	SRP045129	Zhou *et al*. (2015)
Enrei (cultivar)	Reference‐based assembly	GCA_001269945.2	Shimomura *et al*. (2015)
28 Brazilian soybean	Re‐sequencing	PRJNA294227	dos Santos *et al*. (2016)
404 fully domesticated; 36 semi‐domesticated; 72 non‐domesticated	Re‐sequencing		Han *et al*. (2016)
7 wilds; 43 landraces; 56 cultivars	Re‐sequencing	SRP062245	Valliyodan *et al*. (2016)
291 landraces; 278 cultivars	Re‐sequencing	PRJCA000205	Fang *et al*. (2017)
102 cultivars	Re‐sequencing	SRP094720	Torkamaneh *et al*. (2018)
Zhonghuang 13 (cultivar)	De novo sequencing and assembly	CRA001007	Shen *et al*. (2018a)
Zhonghuang 13 (cultivar)	De novo sequencing and assembly	CRA001810	Shen *et al*. (2019)
W05 (wild)	De novo sequencing and assembly	SRP158454	Xie *et al*. (2019)
1 wild; 2 cultivars	De novo sequencing and assembly	GCA_002907465.1;GCA_002905335.1 & PRJNA48389	Valliyodan *et al*. (2019)
3 wilds; 9 landraces; 14 cultivars	De novo sequencing and assembly	PRJCA002030	Liu *et al*. (2020f)
177 landraces; 21 breeding lines	Re‐sequencing	PRJDB7281	Kajiya‐Kanegae *et al*. (2021)
134 cultivars	Re‐sequencing	SRP062560	Qi *et al*. (2021)

In addition to the progresses in genomics, studies on soybean transcriptomics, epigenetics and proteomics were also explored. Transcriptome of different tissues from different developmental stages illuminated gene expression profiling at a whole‐genome level (Severin *et al*., 2010; Wang *et al*., 2014b). Integration of the gene co‐expression network from RNA‐seq data of 1978 samples with previously reported QTLs identified a candidate gene that may control flowering time in soybean (Shen *et al*., 2018a). Meanwhile, large numbers of differentially expression genes (DEGs) that may be related to stresses were identified using the transcriptome data from plants subjected to stress treatments, such as drought, flooding, salt or heat (Chen *et al*., 2016; Liu *et al*., 2019; Wang *et al*., 2018a; Xu *et al*., 2018). These candidate genes provide clues for further functional study. Recently, 1298 publicly available soybean transcriptome datasets were combined to generate a comprehensive atlas of expression, which can be accessed through the website http://venanciogroup.uenf.br/cgi‐bin/gmax_atlas/index.cgi (Machado *et al*., 2020).

DNA methylation profiling analyses revealed that hypomethylation could affect the expression of neighbouring genes (Song *et al*., 2013b). Kim *et al*. (2015) found that CG body‐methylated genes were abundant in duplicated genes that exhibited higher expression level than single copy genes. Moreover, methylation changes proximal to the transposable elements (TEs) were associated with the divergence of expression profiles of duplicated genes (El Baidouri *et al*., 2018; Hossain *et al*., 2017). Shen *et al*. (2018b) investigated the variation of DNA methylation during soybean domestication and improvement and found that differentially methylated regions that are not associated with any genetic variation were enriched in carbohydrate metabolism pathways. It was found that DNA demethylation/methylation also plays critical roles in stress responses, such as continuous cropping (Liang *et al*., 2019), salinity stress (Song *et al*., 2012) and cyst nematode infection (Rambani *et al*., 2020).

Using proteomics approaches, numbers of genes response to various stresses were identified (Khan and Komatsu, 2016; Khatoon *et al*., 2012; Komatsu *et al*., 2010; Komatsu *et al*., 2017; Komatsu *et al*., 2011; Sobhanian *et al*., 2010; Wang and Komatsu, 2018). Similarly, the protein changes through different developmental stages were also investigated (Agrawal *et al*., 2008; Arai *et al*., 2008). Along with the research progresses in proteomics, several proteomics databases were provided. The Soybean Proteome Database (SPD) stores soybean proteomics data obtained from both gel‐based and gel‐free techniques (Komatsu *et al*., 2017; Ohyanagi *et al*., 2012). A user‐intuitive database (http://oilseedproteomics.missouri.edu) stores the expression profile data for proteomics research on soybean and other oilseeds plant (Agrawal *et al*., 2008).

## Germplasm resources and databases

Soybean is rich in germplasm resources that carry large amounts of variations. Over the long history of soybean cultivation, more than 60 000 accessions have been developed (Carter *et al*., 2004; Wilson, 2008). In China, more than 40 000 soybean accessions were collected from all over the nation and stored in the Chinese Crop Germplasm Centre. In the United States, over 20 000 accessions were collected from around the world and stored at the U.S. Department of Agriculture (Song *et al*., 2015). Investigations of the population structure and genetic diversity of the core collections suggested that the accessions exhibit geographical patterns (Haupt and Schmid, 2020; Li *et al*., 2008).

Mutant populations are important in facilitating gene cloning and functional analysis. Through fast neutron, gamma radiation and ethylmethane sulphonate (EMS) mutagenesis, several soybean mutant populations had been constructed (Bolon *et al*., 2014; Espina *et al*., 2018; Li *et al*., 2017e; Tsuda *et al*., 2015). These populations enabled the identification of some genes responsible for important traits (Anderson *et al*., 2019). For example, using TILLING, Liu *et al*. (2012) identified a soybean cyst nematode‐related gene, *GmSHMT*, at the *Rhg4* locus. Dobbels *et al*. (2017) identified an oil biosynthesis‐related gene (*KASI*) through forward screening of a soybean fast neutron mutant population. Reinprecht *et al*. (2009) found that mutations in two *Fad3* genes lead to a low level of linolenic acid in the EMS mutant *RG10*.

Several soybean databases related to soybean genomics, transcriptomics, proteomics and germplasm analyses have been developed. These freely available databases include SoyBase, a database for genetics and genomics from USDA‐ARS (Brown *et al*., 2020; Grant *et al*., 2010); SoyTEdb, a database of transposable elements (Du *et al*., 2010a); SoyNet, a database for co‐functional networks (Kim *et al*., 2017); SoyProDB: a database for seed proteins (Tavakolan *et al*., 2013); SoyProLow: a database for low‐abundance proteins (Tavakolan *et al*., 2014); and SoyKB, a database for translational genomics and molecular breeding (Joshi *et al*., 2014; Table 2). These databases provide valuable platforms for soybean research.

**Table 2:** Soybean databases

Database	URL	Description	Reference
Soybean gene expression atlas	http://www.soybase.org/soyseq	a database of soybean 14 tissues specific gene expression	Severin *et al*. (2010)
Soybean cDNA sequenced	http://digbio.missouri.edu/soybean_atlas	a cDNA database of soybean developmental tissues specifically in root hair and meristem	Libault *et al*. (2010)
SoyNet	http://www.inetbio.org/soynet	a database for network‐based functional predictions	Kim *et al*. (2017)
Soybean transcriptome data	http://venanciogroup.uenf.br/cgi‐bin/gmax_atlas/index.cgi	a database of 1,298 publicly available soybean transcriptome	Machado *et al*. (2020)
Proteomics of oilseeds	http://oilseedproteomics.missouri.edu	expression profile data for proteomics research on soybean and other oilseeds plants	Agrawal *et al*. (2008)
Soybean Proteome Database SPD	http://proteome.dc.affrc.go.jp/Soybean/	a database of soybean proteomics	Ohyanagi *et al*. (2012); Komatsu *et al*. (2017)
SoyBase	http://www.soybase.org	a database of soybean genetics and genomics	Grant *et al*., 2010; Brown *et al*. (2020)
SoyTEdb	http://www.soytedb.org	a database of soybean transposable elements	Du *et al*. (2010a)
SoyProDB	http://bioinformatics.towson.edu/Soybean_Seed_Proteins_2D_Gel_DB/Home.aspx	a database for soybean seed proteins	Tavakolan *et al*. (2013)
SoyProLow	http://bioinformatics.towson.edu/Soybean_low_abundance_proteins_2D_Gel_DB/Gel1.aspx.	a database for soybean low abundant proteins	Tavakolan *et al*. (2014)
SoyKB	http://soykb.org	a database of soybean translational genomics and for soybean molecular breeding	Joshi *et al*. (2014)

## Genes responsible for important agronomic traits

In the past decade, great efforts have been made to identify the genetic loci and the genes responsible for different agronomic traits, including yield, seed quality, stress, development and domestication. The efforts led to the identification of hundreds of QTLs (Hacisalihoglu *et al*., 2018), part of which have been integrated into the Soybase database (https://www.soybase.org/). Despite numerous QTLs were identified, only small number of responsible genes for these QTLs have been cloned, leaving most of them need to be cloned and functionally characterized. Here, we focus on a review of the genes that have been functionally validated (Table S1).

### Yield components

Yield is one of the breeding priorities for soybean. Soybean yield is determined mainly by plant architecture, seed weight and size, and seed number per pod. In the past decade, several key genes controlling yield and related traits were cloned.

#### Plant architecture

Plant architecture is a key trait that significantly affects the yield of soybean. *Dt1* and *Dt2* are two key genes for plant height and growth habits (Liu *et al*., 2010; Ping *et al*., 2014; Tian *et al*., 2010). Analyses of population genetics suggested that *Dt1*, which is a homolog of *Arabidopsis Terminal Flower 1* (*TFL1*), underwent artificial selection to create a determinate growth habit at early stages of landrace dissemination (Tian *et al*., 2010). *Dt2* encodes a MADS‐domain factor (Ping *et al*., 2014) that could bind the promoter of *Dt1* and repress its transcription (Liu *et al*., 2016b). Further functional investigation showed that Dt2 was a direct activator or repressor of the precursors of eight miRNAs whose target genes were associated with meristem maintenance, flowering time, stomatal density, water‐use efficiency (WUE) and/or stress responses (Zhang *et al*., 2019a). Other genes regulating plant architecture have also been characterized, such as *SQUAMOSA‐Promoter Binding Protein‐Like* (*SPL*) and *Late Elongated Hypocotyl* (*LHY*). The *spl9a/spl9b‐1/spl9c/spl9d* homozygous quadruple mutant plants had more branches than WT (Bao *et al*., 2019). *SPL* was regulated by miR156 to affect plant architecture in soybean (Sun *et al*. 2019; Wang and Wang 2015). When miR156b was overexpressed, the transgenic lines increased yield per plant by 46%‐63%, which was resulted from improved long branches number, nodes and pods number, 100‐seed weight (Sun *et al*., 2019). In soybean, the quadruple mutant of *GmLHY* displayed reduced plant height and shortened internodes (Cheng *et al*., 2019). Recently, *GmMYB14* were characterized as an important factor to regulate plant architecture, high‐density yield and drought tolerance through the brassinosteroid (BR) pathway in soybean (Chen *et al*., 2021).

#### Seed size

In *Arabidopsis*, *P450/CYP78A* gene family had been found to control seed size (Fang *et al*., 2012; Wang *et al*., 2008). The orthologs of *P450/CYP78A* in soybean, including *GmCYP78A10*, *GmCYP78A72*, *GmCYP78A57* and *GmCYP78A70*, showed conserved function to control seed size (Du *et al*., 2017; Wang *et al*., 2015b; Zhao *et al*., 2016). The genes *BIG SEEDS1* (*BS1*) (Ge *et al*., 2016), *GmKIX8‐1* (Nguyen *et al*., 2020), *GmCIF1* (Tang *et al*., 2017), *GmPSKγ1* (Yu *et al*., 2019), *WRKY15a* (Gu *et al*., 2017) and *phosphatase 2C‐1* (*PP2C‐1*) (Lu *et al*., 2017b) were also involved in seed development in soybean. Furthermore, *SoyWRKY15a* and *PP2C‐1* were found to have undergone artificial selection during soybean domestication (Gu *et al*., 2017; Lu *et al*., 2017b).

#### Flowering

Soybean is a short‐day and photoperiod sensitive plant (Miranda *et al*., 2020). Genetic analyses of natural variants have identified 12 maturity loci—*E1* to *E11* and *J*—that control flowering time and maturity. In the past decade, significant progress has been made in soybean flowering gene identification and functional characterization (Lin *et al*., 2020b).

*E1* encodes a soybean‐specific transcription factor that contains a plant‐specific B3 domain (Xia *et al*., 2012). *E1* could repress flowering under long‐day (LD) conditions, while its leaky allele (*e1‐as*) and null alleles (such as *e1‐fs* and *e1‐nl*) caused earlier flowering (Xia *et al*., 2012; Xu *et al*., 2013a; Yasutaka *et al*., 2013). *E2* encodes a homolog of *Arabidopsis GIGANTEA (GI)*, which is a component of the circadian clock (Watanabe *et al*., 2011). The functionally dominant *E2* delayed flowering time, while the recessive *e2* induced flowering (Watanabe *et al*., 2011). *E3* and *E4* were found to be functionally redundant and encode *GmPHYA3* (Watanabe *et al*., 2009) and *GmPHYA2* (Liu *et al*., 2008; Yasutaka *et al*., 2013), respectively, which are homologues of the photoreceptor phytochrome A (PHYA) (Franklin and Quail, 2010). *E3* and *E4* were involved in the control of flowering under LDs with high red:far‐red (R:FR) quantum ratios and low R:FR ratios, respectively (Cober *et al*., 1996; Cober and Voldeng, 2001). *J* is an important locus for the adaptation of soybean to lower latitudes. *J* was found to encode an EARLY FLOWERING 3 (ELF3) homolog (Fang *et al*., 2020; Lu *et al*., 2017a; Yue *et al*., 2017). A pair of pseudo‐response‐regulators (PRRs; named *Tof11* and *Tof12*) were reported to contribute to changes in flowering and early maturity in soybean (Day *et al*., 2010; Gong, 2020; Li *et al*., 2020a; Lu *et al*., 2020b; Wang *et al*., 2020c).

Other genes have also been reported to function in regulating flowering in soybean, particularly genes homologous to flowering‐related genes from other species. *FLOWERING LOCUS T (FT)* is required for flowering and widely conserved among plant species. In soybean, *10 FT/TSF* homologs were identified. These *FT/TSF* homologs had divergence functions, and their natural variation might be associated with soybean adaptation to different environments (Jiang *et al*., 2019; Kong *et al*., 2010; Wu *et al*., 2017). Overexpression of *GmFT2a/E9* or *GmFT5a* promoted flowering in soybean, whereas overexpression of *GmFT1a* or *GmFT4* suppressed flowering (Cai *et al*., 2018; Cai *et al*., 2020b; Chen *et al*., 2020b; Kong *et al*., 2010; Liu *et al*., 2018; Nan *et al*., 2014; Takeshima *et al*., 2016; Zhai *et al*., 2014a). *FT4* is a candidate gene for the *E10* locus (Samanfar *et al*., 2017). Although *FT2a* and *FT5a* showed similar functions in inducing flowering, *FT5a* was more effective than *FT2a* in the post‐flowering termination of stem growth (Takeshima *et al*., 2019). Further investigation showed that several floral genes, such as *GmAP1*, *GmSOC1* and *GmLFY*, were significantly induced by *GmFT2a/E9* and *GmFT5a* (Nan *et al*., 2014). Overexpression of *GmAP1a*, one of the homologs of *Arabidopsis APETALA1* (*AP1*) which functions as a class A gene in the ABCDE model of flowering, resulted in early flowering and reduced plant height compared with the wild type under short‐day (SD) conditions (Chen *et al*., 2020c). GmFDL19, which is a bZIP transcription factor, interacted with GmFT2a/E9 and GmFT5a. Overexpression of *GmFDL19* in soybean resulted in early flowering through the regulation of the expression of *GmAP1a* (Nan *et al*., 2014). *GmGBP1* functioned as a positive regulator upstream of *GmFT2a/E9* and *GmFT5a* to activate the expression of *GmFULs* to promote flowering under LDs (Zhao *et al*., 2018).

In the photoperiod‐controlled flowering pathway, the circadian clock‐regulated gene *CONSTANS (CO)* is crucial for the induction of the *FT* gene (Turck *et al*., 2008). The soybean genes *GmCOL1a/GmCOL1b* showed high sequence homology to *AtCO*. The late‐flowering phenotype of the *Arabidopsis co‐1* mutant was fully complemented by overexpression of *GmCOL1a* or *1b*, suggesting that they function similarly to *AtCO* at the protein level (Wu *et al*., 2014). Further analysis showed that *GmCOL1a* controlled flowering time by suppressing the expression of *GmFT2a/E9* and *GmFT5a* under LDs. In addition, *GmCOL1a/1b* was up‐regulated by *E1*, *E2*, *E3* and *E4* (Cao *et al*., 2015a). It was found that overexpression of *GmAGL1*, which encodes a MADS‐box protein, promoted flowering, maturity and led to a smaller floral organ (Chi *et al*., 2017; Zeng *et al*., 2018). Moreover, several miRNA families were also demonstrated to play important roles in controlling flowering in soybean. Overexpression of *MIR156b*, which is involved in the PHYA‐mediated photoperiod response pathway, delayed flowering under LDs (Cao *et al*., 2015b). In *Arabidopsis*, miR156 regulated the expression of miR172 via *SPL9* (Wu *et al*., 2009). In soybean, miR156b down‐regulated *MIR172* and *SPL9* and up‐regulated the miR172 target gene *GmTOE4a* in soybean (Cao *et al*., 2015b). In addition, *E2* could promote the maturation of *MIR172a* via increasing the expression of DICER‐LIKE 1 and SERRATE homologs (Wang *et al*., 2016b).

**Figure 2:**
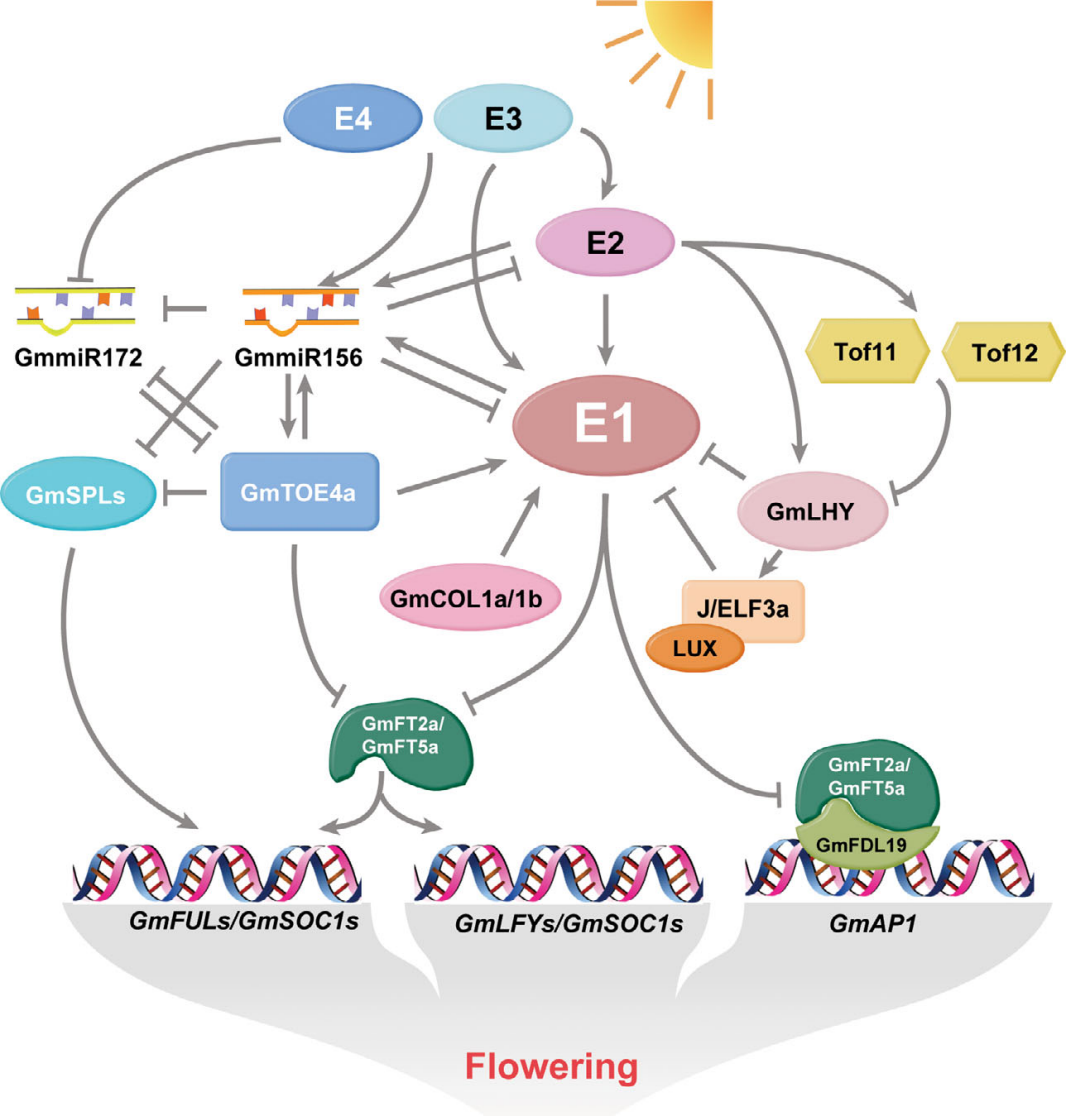
Proposed molecular regulation network of flowering in soybean. Soybean is a short‐day flowering plant. E3 and E4 mediate flowering by responding to the ratios of red (R) and far‐red (FR) light. Under long days (LDs), E3 and E4 induce the expression of *E1*. GmTof11 and GmTof12 inhibit the expression of *GmLHYs* by binding to their promoters. GmLHY proteins directly bind to the promoter region of J to induce its expression and bind to the promoter of E1 to suppress its transcription. LUX can physically interact with GmELF3a/b to repress *E1* expression. E1 inhibits the expression of the flowering‐inducing factors *GmFT2a* and *GmFT5a*. This suppresses the expression of floral identity genes (*GmAP1, GmSOC1s, GmLFYs, GmFULs)*. As a result, flowering is delayed under LDs. Under SDs, the induction of *E1* is decreased, which releases the transcriptional suppression of *GmFT2a* and *GmFT5a*, promoting flowering. The E2 and miRNA‐dependent flowering regulation modules are also influenced by photoperiod under PHYA mediation (E3 and E4). GmCOL1a/1b and GmTOE4a inhibit flowering via down‐regulation of *GmFT2a* and *GmFT5a* expression. *GmTOE4a* expression is possibly controlled by miR172, which is under the control of miR156b. miR156b may delay flowering by negatively regulating the *GmSPLs*.

Functional investigations have demonstrated that the above genes are involved in a complicated PHYA‐mediated photoperiod response pathway that regulates flowering time in soybean (Kong *et al*., 2010; Li *et al*., 2020a; Lu *et al*., 2020b; Lu *et al*., 2017a; Xia *et al*., 2012; Xu *et al*., 2015). In Figure 2, we summarized the current understanding of this flowering time regulation under LDs. Briefly, *E3* and *E4* were up‐regulated *Tof11* and *Tof12* expression. Then, Tof11 and Tof12 proteins physically could bind to the promoters of the *GmLHY* genes to suppress their expression, which suppressed their subsequent induction of *J*. At low levels, J is not able to suppress the transcription of *E1*. The abundance of *E1* decreases the expression of *GmFT2a/E9* and *GmFT5a*, ultimately resulting in delayed flowering and later maturity (Figure 2). Recently, it was found that GmLUX2, an ortholog of the *Arabidopsis* Evening Complex (EC) component LUX ARRHYTHMO (LUX), could physically interact with GmELF3a/b to regulate the expression of several circadian clock‐associated genes and directly repress *E1* expression (Fang *et al*., 2021a) (Figure 2).

#### Others

Other genes that control yield‐related developmental traits were also identified. *Ln*, which encodes a C2H2 zinc finger transcription factor, was found to be a key regulator of seed number per pod (Fang *et al*., 2013; Jeong *et al*., 2011; Jeong *et al*., 2012). *GmILPA1*, which encodes an APC8‐like protein, was found to control petiole angle (Gao *et al*., 2017). *Chicken Toes‐Like Leaf and Petalody Flower* (*CTP*) was found to be a novel regulator that controlled leaf and flower development in soybean. Mutation of *CTP* resulted in leaf defects and additional flower parts developing into petals (Zhao *et al*., 2017). It was found that *GmCRY1s* could modulate gibberellin metabolism during the response to reduced blue light, affecting yield in a shaded environment (Lyu *et al*., 2021).

Several genes controlling senescence are also identified. *D1* and *D2*, homologs of *STAY‐GREEN* (*SGR*), and *GmCHLI*, a subunit of magnesium (Mg)‐chelatase, regulated chlorophyll degradation in soybean (Fang *et al*., 2014; Li *et al*., 2016a; Slattery *et al*., 2016). The maternally inherited stay‐green gene *cytG* was found to encode the chloroplast protein PsbM (Kohzuma *et al*., 2017). Under blue light, cryptochrome2 (CRY2a) interacts with the soybean basic helix‐loop‐helix transcription activator CIB1 to regulate leaf senescence in soybean (Meng *et al*., 2013).

### Seed composition

Protein, oil and isoflavone content are the main soybean seed quality traits and are determined via interaction between quantitative loci and the environment. Seed storage proteins usually showed negative correlation with oil content (Chaudhary *et al*., 2015) and seed yield (Bandillo *et al*., 2015; Chung *et al*., 2003; Zhang *et al*., 2018). Besides, the contents of soluble sugar, mineral nutrition and amino acid are also important for seed quality. Hundreds of QTLs that affected soybean seed quality have been identified using both pedigree‐based and association mapping approaches. Some of this information has been integrated into Soybase (https://www.soybase.org/).

#### Oil content and fatty acid

Triacylglycerols (TAGs) are the main components of soybean seed oil. Lines carrying knockdowns of four TAG lipase‐encoding genes, namely *GmSDP1‐1*, *GmSDP1‐2*, *GmSDP1‐3* and *GmSDP1‐4*, have significantly increased seed oil content (Kanai *et al*., 2019). Overexpression of the phospholipid diacylglycerol acyltransferase *GmPDAT* increased seed oil and also altered size‐related traits (Liu *et al*., 2020a; Liu *et al*., 2020b). A comprehensive assessment of the gene co‐expression networks showed that *GA20 oxidase* (*GA20OX*) and *nuclear transcription factor Y subunit alpha* (*NFYA*) are two key drivers of seed traits. Overexpression of *GA20OX* and *NFYA* enhanced seed size and weight and oil content, respectively (Lu *et al*., 2016). It was found that oleosin‐encoding *GmOLEO1* was predominantly expressed during seed maturation, and overexpression of *GmOLEO1* significantly increased seed oil content by 10.6% (Zhang *et al*., 2019b). Overexpression of *GmWRI1a*, an APETALA2/ethylene responsive element‐binding protein (AP2/EREBP) encoding gene, under the control of a seed‐specific promoter significantly increased total oil and fatty acid content and also changed fatty acid composition (Chen *et al*., 2018b). Genetic modification of the fatty acid desaturase *FAD* significantly changes the composition of polysaturated and polyunsaturated fatty acids (Al Amin *et al*., 2019; Andreu *et al*., 2010; Combs and Bilyeu, 2019; Haun *et al*., 2014; Lakhssassi *et al*., 2017; Lim *et al*., 2014; Wagner *et al*., 2011). Genetic modification of *DGAT*, which encodes a type 1 diacylglycerol acyltransferase, increased the oil content and decreases the soluble carbohydrate content in soybean seeds (Roesler *et al*., 2016).

Several transcription factors were also found to be associated with seed quality. Overexpression of transcription factors *GmbZIP123*, *GmMYB73*, *GmZF351*, *GmZF392* and *GmWRI1b* enhanced lipid content (Guo *et al*., 2020; Li *et al*., 2017b; Liu *et al*., 2014b; Song *et al*., 2013a). Further functional investigation showed that the enhancement of oil content by GmMYB73 was through interaction with GL3 and EGL3 and the suppression of GL2, a negative regulator of oil accumulation (Liu *et al*., 2014b). *GmZF351* was more highly expressed in cultivated soybeans compared with wild soybeans, and this was due to a mutation of its promoter, which had undergone artificial selection during soybean domestication (Li *et al*., 2017b). GmZF392 physically interacted with GmZF351, and both *GmZF392* and *GmZF351* could be further induced by *GmNFYA* (Lu *et al*., 2021).

#### Isoflavone

Isoflavone content is highly influenced by the genes involved in chalcone synthesis, including chalcone synthase, chalcone reductase and chalcone isomerase (Dastmalchi *et al*., 2016). Among 11 chalcone reductase (CHR) proteins of soybean, CHR5 was found to interact with a 2‐hydroxyisoflavanone synthase (IFS) isozyme and most correlated with the distribution patterns of 5‐deoxyisoflavonoids, indicating that it may play an important role in the isoflavone pathway (Mameda *et al*., 2018). In soybean, some prenyltransferase‐encoding genes exhibited isoflavonoid‐specific patterns (Sukumaran *et al*., 2018). When the gene encoding carotenoid cleavage dioxygenase 4 (*GmCCD4*) was knocked out, the loss‐of‐function lines showed increased carotenoid content and yellow flowers (Gao *et al*., 2021). Several independent studies demonstrated that the *MYB* gene family played important roles in affecting isoflavone synthesis, which may involve a pathway that consists of 14‐3‐3s and CHS proteins (Chu *et al*., 2017; Li *et al*., 2012; Pandey *et al*., 2014; Yi *et al*., 2010). Recently, it was reported that selection of a class B heat‐shock factor, *HSFB2b*, in soybean domestication promoted flavonoid biosynthesis and enhanced salt tolerance (Bian *et al*., 2020). An analysis combining GWAS and QTLs identified *GmMPK1*, which encodes a mitogen‐activated protein kinase, as one of the candidate genes responsible for isoflavone content (Wu *et al*., 2020).

#### Protein content, amino acid and others

Compared with genes for oil content, fewer genes controlling protein content or amino acid have been functionally identified (Krishnan and Jez, 2018). It was found that MGL, a putative methionine γ‐lyase, may be responsible for the accumulation of S‐methylmethionine in soybean seed (Teshima *et al*., 2020). Overexpression of the cytosolic isoform of O‐acetylserine sulfhydrylase (OASS) and the plastid ATP sulfurylase isoform 1 improved the cysteine‐rich proteins and sulphur amino acid content in transgenic soybean, independently (Kim *et al*., 2012b; Kim *et al*., 2020). *Rab5a*, a small GTPase‐encoding gene, was reported to be involved in post‐Golgi trafficking of storage proteins in developing soybean cotyledons (Wei *et al*., 2020b). Recently, it was found that SWEET sugar transporters played important roles in soybean seed quality through effects on the contents of both oil and protein. *GmSWEET15* mediated sucrose export from endosperm to early embryo (Wang *et al*., 2019d). *GmSWEET10a* and *GmSWEET10b* determined oil and protein contents and seed size simultaneously in soybean through affecting sugar allocation from seed coat to embryo (Miao *et al*., 2020; Wang *et al*., 2020e; Zhang *et al*., 2020c).

### Stress resistance

Abiotic and biotic stresses greatly affect soybean yield and quality (Anderson *et al*., 2019). Drought and salinity can reduce soybean yield by 40% through negative impacts on growth, nodulation, flowering, seed quality and seed quantity (Anderson *et al*., 2019; Papiernik *et al*., 2005; Specht *et al*., 1999). Stress (including drought, salt, temperature stress, flooding and disease) has been intensely studied in soybean (Kunert *et al*., 2016; Li *et al*., 2021; Li *et al*., 2014a; Phang *et al*., 2008; Ramesh *et al*., 2019; Robison *et al*., 2019; Shu *et al*., 2020; Whitham *et al*., 2016; Widyasari *et al*., 2020).

#### Drought

Several transcription factors were reported to play important roles in soybean drought tolerance, such as *WRKY* and *NAC* gene families. *GmWRKY54* increased drought tolerance by activating genes in the abscisic acid and Ca^2+^ signalling pathways (Wei *et al*., 2019). Overexpression of *WRKY20* from a wild soybean significantly enhanced drought tolerance in *Arabidopsis* (Luo *et al*., 2013). *GmNAC8* overexpression and knockout transgenic lines exhibit significantly higher and lower drought tolerance, respectively, suggesting that *GmNAC8* is a positive regulator of drought tolerance (Yang *et al*., 2020).

U‐box (PUB) proteins function as E3 ligases in plants. When *GmPUB6* was overexpressed in *Arabidopsis*, the transgenic lines exhibited decreased plant survival under drought stress condition (Wang *et al*., 2020d). Several reports suggested that circadian clock genes are also involved in drought tolerance, such as the *GmLHYs* and *GmLCL*, could affect drought tolerance (Wang *et al*., 2021a; Yuan *et al*., 2021). It is reported that many clock genes respond to both flooding and drought, with the expression patterns of some genes shifting in amplitude and phase (Marcolino‐Gomes *et al*., 2014; Syed *et al*., 2015).

Other genes that affect drought tolerance in soybean have also been identified. For instance, the genes responsible for pubescence density, *Pd1*, *P1*, *Ps* and *Pd2*, were also found to be involved in drought tolerance in soybean (Liu *et al*., 2020c; Pfeiffer *et al*., 2003). Overexpression of the *LOS5/ABA3* gene, which encodes a molybdenum cofactor sulfurase, using a constitutive expression promoter in soybean enhanced drought tolerance and increased seed yield by at least 21% under drought stress (Li *et al*., 2013a). Drought tolerance in soybean through foreign gene transformation was also achieved, such as the overexpression of rice *cystatin oryzacystatin I* (*OCI*), *Arabidopsis DREB1A*, and the sunflower transcription factor *HB4* (Quain *et al*., 2014; Rakocevic *et al*., 2018; Ribichich *et al*., 2020).

#### Salt

Stresses, such as salinity, osmotic stress, imbalance of ions, ion toxicity and excessive reactive oxygen, have significant effects on the growth of plant (Ruiz‐Lozano *et al*., 2012). Under high salinity, plants slow down photosynthesis and ramp up sugar catabolism to provide extra energy for survival (Liu *et al*., 2019). Balancing the ions and reducing the ion toxicity are the keys to enhance the salt resistant. Through forward genetic approaches, two research groups independently found that *GmSALT3*, which encodes a cation/H+ exchanger, could limit the accumulation of sodium ions (Na^+^) in shoots and enhance salt tolerance in soybean (Guan *et al*., 2014; Qi *et al*., 2014). Further investigation suggested that *GmSALT3* functioned through exclusion of sodium ions (Na^+^) from the leaf via a root‐derived mechanism and exclusion of chloride ions (Cl^–^) via a shoot‐derived process (Qu *et al*., 2021). *GmCDF1*, encoding a cation diffusion facilitator, could negatively regulate salt tolerance by maintaining K^+^‐Na^+^ homeostasis in soybean (Zhang *et al*., 2019c). *GmAKT1*, a K^+^ transporter encoding gene, located in the plasma membrane and was recently reported to play an important role in soybean salt resistance by regulating the K^+^ uptake and Na^+^/K^+^ balance (Wang *et al*., 2021b).

Other genes that have effects on salt tolerance in soybean were also reported. *GmNAC109* and *SALT INDUCED NAC1* (*GmSIN1*) were found to promote root growth and increase abiotic stress tolerance through up‐regulation of ABA synthesis‐associated and ROS generation genes (Li *et al*., 2019c; Yang *et al*., 2019). The salt‐inducible gene *GmbZIP110* encodes a protein that could bind to the promoters of genes with an ACGT motif and impact the expression of many stress‐related genes and enhance salt tolerance (Xu *et al*., 2016). Overexpression of *GmNAC20* or *GmNAC11* enhanced salt tolerance in transgenic *Arabidopsis* plants (Hao *et al*., 2011). Interestingly, miR172 was also involved in the response to salt stress. Under salinity, plants with hairy roots overexpressing the pre‐miR172a had healthier leaves and larger roots. Further investigation showed that miR172a promoted salt tolerance mainly through cleaving the AP2/EREBP‐type transcription factor *SSAC1* gene, releasing its inhibition on *THI1*, which encodes a positive regulator of salt tolerance (Pan *et al*., 2016). Overexpression of soybean *MIR172c* conferred tolerance to both water deficit and salt stress in transgenic *Arabidopsis* (Li *et al*., 2017d). The plant homeodomain protein GmPHD6 functioned as a histone code reader and interacted with LHP1 to form a transcriptional activator that regulated genes for salt tolerance. Overexpression of *GmPHD6* improved salt tolerance in soybean (Wei *et al*., 2017). *HSFB2b*, a B heat‐shock factor encoding gene, improved salt tolerance by promoting flavonoid biosynthesis. Interestingly, *HSFB2b* had undergone artificial selection during soybean domestication (Bian *et al*., 2020). In addition, mitogen‐activated protein kinase (MAPK), BURP‐domain proteins and NAD(P)H dehydrogenase (NDH) were also found to be involved in salt tolerance in soybean (He *et al*., 2015; Im *et al*., 2012; Wang *et al*., 2012a).

Genes can affect both drought and salt tolerance were also reported. For instance, overexpression of *GmMYB118*, *GmERF135*, *GmCDPK3* improved drought and salt tolerance simultaneously (Du *et al*., 2018; Wang *et al*., 2019a). Interestingly, it was also found that even different members from the same WRKY family having differential effects on abiotic stress tolerance in soybean (Wang *et al*., 2015a; Wei *et al*., 2019; Zhou *et al*., 2008).

#### Minerals

Iron deficiency results in stunting and yield loss and is one of the most common and severe nutritional stresses for soybean (Hacisalihoglu *et al*., 2018). Soybean plants reprogramme metabolism under iron deficiency (Chu *et al*., 2019). Moreover, the circadian clock was highly responsive to iron deficiency (Li *et al*., 2019b). Several genes that may be involved in iron deficiency regulation were identified. Silencing of *GmRPA3*, which encodes subunit 3 of replication protein A, showed reduced iron deficiency chlorosis (IDC) and increased chlorophyll content under iron‐deficient conditions (Atwood *et al*., 2014). It was found that the physical interaction between two soybean bHLH factors, GmbHLH57 and GmbHLH300, was important for Fe homeostasis (Li *et al*., 2018a). Genetic variation of the genes encoding a Fe deficiency‐induced transcription factor and a Fe/Zn‐regulated transporter may be responsible for variation of iron use efficiency among different soybean accessions (Liu *et al*., 2020f; Peiffer *et al*., 2012).

Several reports have focused on the investigation of phosphorus‐use efficiency (PUE). *GmACP1* encodes an acid phosphatase and is a candidate for a PUE locus identified via GWAS. Overexpression of *GmACP1* in soybean hairy roots significantly increased PUE (Zhang *et al*., 2014). Under Pi starvation, the expression of *GmPT1*, *GmPHR25* and *CWPs* was induced, indicating they may be involved in PUE regulation (Song *et al*., 2014; Wu *et al*., 2018; Xue *et al*., 2017). The functions of genes in the *GmALMT* family were also investigated, and GmALMT5 was found to enhance utilization of soluble P under P‐limited conditions (Peng *et al*., 2018). Moreover, ethylene may also affect PUE. Up‐regulating *GmETO1*, which encodes ethylene‐overproduction protein 1, could significantly improve phosphorus uptake and use efficiency in soybean (Zhang *et al*., 2020d). A single point mutation in *GmHMA3*, which encodes a heavy‐metal transporter, may be responsible for differential cadmium (Cd) translocation and accumulation in the seeds of different soybean accessions (Wang *et al*., 2012b). Further, hydrogen sulphide affected aluminium and nitrogen assimilation (Wang *et al*., 2019b; Zhang *et al*., 2020e).

#### Pathogens

Soybean cyst nematode (SCN) is the most devastating soybean pathogen in the United States, with yield losses ranging from 1.9 to 3.5 million tonnes per year (Liu *et al*., 2015; Wrather and Koenning, 2006). SCN infection induced a large number of genes related to cell wall modification, stress response, defence and signal transduction (Kandoth *et al*., 2011; Rambani *et al*., 2015; Tucker *et al*., 2011). The proteins potentially related to SCN were collected in the database SCNProDB (Natarajan *et al*., 2014).

*Rhg1* and *Rhg4* are two major QTLs/genes conveying SCN resistance (Liu *et al*., 2012; McHale *et al*., 2012; Mitchum, 2016). *Rhg4* encodes a serine hydroxymethyltransferase (SHMT), which mediated a novel plant resistance mechanism against a pathogen (Liu *et al*., 2012). In the *Rhg1* region, the copy number of three genes, *Glyma18g02580*, *Glyma18g02590* and *Glyma18g02610*, was associated with SCN resistance (Cook *et al*., 2014; Cook *et al*., 2012; Lee *et al*., 2016). Further investigation demonstrated that the repeated copies of a gene encoding atypical α‐soluble N‐ethylmaleimide‐sensitive factor (NSF) attachment protein (α‐SNAP) were the most likely candidate for conferring resistance to SCN (Liu *et al*., 2017; Patil *et al*., 2017). In *Rhg1*(+) germplasm, analysis of an unusual NSF allele [*Rhg1*‐associated NSF on chromosome 07; NSF (RAN07)] revealed that NSF (RAN07) exhibited stronger *in vitro* binding with *Rhg1* resistance‐type α‐SNAPs, suggesting that an atypical co‐evolution of the soybean SNARE‐recycling machinery balances the acquisition of an otherwise disruptive housekeeping protein, enabling a valuable disease resistance trait (Bayless *et al*., 2018).

Resistance to SCN involves salicylic acid. Overexpression of a salicylic acid methyltransferase gene conferred resistance to SCN (Lin *et al*., 2016; Lin *et al*., 2013). The concentration of 1‐aminocyclopropane‐1‐carboxylic acid (ACC) and expression of ACC synthase were both higher in SCN‐colonized root pieces and root tips than in other parts of the root (Tucker *et al*., 2010). Moreover, *CLE* (encodes an extracellular protein), *GmAFS* (encodes one member of the soybean terpene synthase gene family), *MIR396* and *t*‐*SNAREs* also respond to SCN infection (Dong *et al*., 2020; Guo *et al*., 2015; Lin *et al*., 2017; Noon *et al*., 2019; Noon *et al*., 2016). Along with SCN infection, variation of DNA methylation in some genomic regions associated with changes in gene expression (Rambani *et al*., 2020).

A report on the dissection of the QTL for southern root‐knot nematode (RKN) resistance in soybean identified three QTL and mapped the major QTL to a 29.7‐kb region on chromosome 10 (Xu *et al*., 2013b). In addition, there are several reports on soybean resistance to other pests. For instance, defence against *Anticarsia gemmatalis* larvae was modulated by solar UV‐B radiation and ethylene (Dillon *et al*., 2018). Recently, a gene encoding a VQ motif‐containing protein, *GmVQ58*, was found to enhance soybean resistance to the common cutworm (Li *et al*., 2020c).

Asian soybean rust (ASR), caused by the obligate biotrophic fungus *Phakopsora pachyrhizi*, and is one of the most economically important diseases for soybean. Through *in vivo* assessment by Mach‐Zehnder double‐beam interferometry, Loehrer *et al*. found that *P. pachyrhizi* might be able to forcefully invade a wide range of plants through appressorial turgor pressure (Loehrer *et al*., 2014). Both a UDP‐glucosyl transferase and phenylpropanoid metabolism are essential for the infection of *P. pachyrhizi* (Beyer *et al*., 2019; Langenbach *et al*., 2013), while coumarin could be used as a natural fungicide against ASR (Beyer *et al*., 2019; Langenbach *et al*., 2013). Evolutionary analyses indicated that some disease resistance genes have conserved function between soybean and other species, indicating that genes identified from other species could be used in soybean disease resistance (Ashfield *et al*., 2014; Okutani *et al*., 2020; Redditt *et al*., 2019; Wang *et al*., 2014a; Wei *et al*., 2020a). When the gene *CcRpp1* (*Cajanus cajan Resistance against Phakopsora pachyrhizi 1*) from pigeon pea or *NHR*‐linked genes from *Arabidopsis* were transferred to soybean, the transgenic lines exhibited resistance to *P. pachyrhizi* (Kawashima *et al*., 2016; Langenbach *et al*., 2016).

Through GWAS and QTL analyses, several loci associated with pattern‐triggered immunity (Valdés‐López *et al*., 2011), sudden death syndrome resistance (Zhang *et al*., 2015) and white mould (Zhao *et al*., 2015b) were identified. Genetic mapping suggested that *STAY‐GREEN* genes may be involved in sudden death syndrome (Chang *et al*., 2019), and *Rsv4*, which encodes an RNase H family protein with dsRNA‐degrading activity, may be responsible for broad‐spectrum mosaic virus resistance (Ishibashi *et al*., 2019). Overexpression of the *HSP40* gene, which encodes a nuclear‐localized, type‐III DnaJ domain‐containing protein, or *GmLMM1*, which encodes a malectin‐like receptor kinase, regulated cell death and disease resistance (Liu and Whitham, 2013; Wang *et al*., 2020a). Overexpression of the plasma membrane protein gene *GmDR1* generated broad‐spectrum immunity (Ngaki *et al*., 2021). Interestingly, genes encoding small peptides, such as *GmSubPep*, encoding a 12‐amino acid peptide (Pearce *et al*., 2010), and *GmPep914*, encoding an 8‐amino acid peptide (Yamaguchi *et al*., 2011), also played important roles in defence against disease. It was recently found that hydroperoxide lyase modulated the defence response and conferred lesion‐mimic phenotype in soybean leaves (Wang *et al*., 2020f).

Phytophthora root and stem rot, caused by *Phytophthora sojae*, is another destructive soybean disease. Overexpression of *GmERF5*, which encodes ethylene response factor 5 (Dong *et al*., 2015), or *GmMYB29A2*, which encodes a glyceollin transcription factor (Jahan *et al*., 2020), significantly enhanced resistance to *P. sojae*. Moreover, overexpression of some microRNAs, such as miR393, could promote soybean defence against *P. sojae* (Wong *et al*., 2014).

Soybean mosaic virus (SMV) is one of the most prevalent viral diseases and could significantly reduce yield losses in soybean. Until now, four dominant SMV resistance loci (*Rsv1*, *Rsv3*, *Rsv4* and *Rsv5*) have been genetically identified (Hayes *et al*., 2000; Jeong *et al*., 2002; Klepadlo *et al*., 2017; Yu *et al*., 1994). Recently, *GmST1*, which encodes a sulfotransferase, was identified as the responsible gene for conferring the resistance to strains G2 and G3 (Zhao *et al*., 2021).

*R* genes specifically activate resistance responses that are effective against diverse pathogens (Zheng *et al*., 2016). In soybean, Kang *et al*. (2012) predicted that the genome contains a total of 319 nucleotide‐binding site/leucine‐rich repeat (NBS‐LRR) *R* genes. The ULP1‐NBS‐LRR protein GmRpp1 confers immunity to *P. pachyrhizi* (Pedley *et al*., 2019). Overexpression of the TIR–NBS–LRR *R* gene *GmKR3* enhanced soybean resistance to several strains of soybean mosaic virus (SMV), which is one of the most prevalent viral diseases and could significantly reduce yield losses in soybean (Xun *et al*., 2019). Silencing of the soybean *NDR1* homologs (*GmNDR1*) showed that they were required for pathogen resistance (Selote *et al*., 2014). *GmMPK4*‐ and *GmMPK6*‐silenced plants displayed strong phenotypes, including induction of PR gene expression and increased SA levels (Liu *et al*., 2014a; Liu *et al*., 2011). Fine‐tuning the expression of pathogen avirulence (Avr) effector genes using genome editing impacted the compatibility of plant disease, which provided clues to improve crop disease resistance (Ochola *et al*., 2020).

#### Other stressors

Salicylic acid (SA) and abscisic acid (ABA) are two important phytohormones for stress resistance. Knockdown of either the phenylalanine ammonia lyase or isochorismate synthase biosynthesis pathway shuts down SA biosynthesis and abrogates pathogen resistance (Shine *et al*., 2016). In soybean, it was found that *ABA‐Sensitive 1* (*GmABAS1*), which encodes a 1R‐subtype of MYB, functioned as a transcriptional repressor that enhances ABA sensitivity (Ku *et al*., 2020). Several transcription factor families were found to be involved in various stress responses. For instance, AP2/ERF‐type transcription factor family members, including *GmERF3* and *GsERF7*, played cardinal roles in regulating resistance to diseases as well as salt and drought (Feng *et al*., 2020; Zhang *et al*., 2009), whereas the DREB1/CBF‐type transcription factors function in heat, drought and cold stresses (Kidokoro *et al*., 2015).

Other genes that response various stresses were also reported. The root‐specific protein kinase‐encoding gene *GmWNK1* and the mitogen‐activated protein kinase‐encoding gene *GmMPK* were found to regulate plant growth and development and, in turn, affect stress responses (Liu *et al*., 2011; Wang *et al*., 2010). Malate exudation mediated by *Gm Representative*, which encodes an expansin, was found to be involved in multiple abiotic stresses (Guo *et al*., 2011). A chaperone binding protein (BiP) functioned as a negative regulator to attenuate stress‐induced cell death (Reis *et al*., 2011). Omega‐3 fatty acid desaturase was found to play an important role in cold response in soybean (Román *et al*., 2012). For competition with weeds, a PIF3‐like protein was identified as a candidate that might manipulate the weed stress response in soybean (Horvath *et al*., 2015).

### Nodulation

Symbiotic nitrogen fixation (SNF) through root nodulation is an important feature of legumes and plays important roles in plant growth. Over the past decade, the genes required for symbiotic nitrogen fixation have been thoroughly investigated, and significant progress has been achieved (Roy *et al*., 2020).

A comprehensive phylogenomic analysis revealed that multiple losses of some key genes, such as *NIN* and *RPG*, were essential for the origin of SNF (Griesmann *et al*., 2018). A global co‐expression analysis suggested that ancient orthologous and duplication events before the origin of legumes had paved the way for nodule formation and nitrogen fixation (Wu *et al*., 2019). Large‐scale transcriptome and metabolome investigations revealed a number of genes and metabolic pathways that are induced or suppressed during nodulation (Agtuca *et al*., 2020; Hayashi *et al*., 2012b; Libault *et al*., 2010). For instance, G protein‐encoding genes and a putative beta‐carotene hydroxylase gene (*GmBCH1*) exhibited significant transcriptional changes in response to rhizobium infection. RNA interference suppression of the genes encoding G protein and *GmBCHs* severely impaired nitrogen fixation as well as nodule development, suggesting they are positive regulators in nodule formation (Choudhury and Pandey, 2013; Kim *et al*., 2013). Furthermore, the G protein cycle was regulated by the activity of phosphorylation‐dependent G protein signalling proteins (Choudhury and Pandey, 2015). The *G‐box Factor 14‐3‐3* genes, *SGF14c* and *SGF14l*, had been shown to function as dimers in soybean nodulation (Radwan *et al*., 2012).

In soybean, the formation of symbiotic root nodules was highly affected by several host genes, referred to as *Rj* or *rj* (Hayashi *et al*., 2012a). *Rj1* and *Rj5* encode putative Nod factor receptors (NFRs) (Hayashi *et al*., 2012a; Indrasumunar *et al*., 2010; Indrasumunar *et al*., 2011), *Rj2*/*Rfg1* encodes a Toll‐interleukin receptor/nucleotide‐binding site/leucine‐rich repeat (TIR‐NBS‐LRR) plant resistance (R) protein (Yang *et al*., 2010), while *Rj7* encodes a nodule autoregulation receptor kinase (Hayashi *et al*., 2012a; Nishimura *et al*., 2002; Searle *et al*., 2003). One report suggested that a gene encoding thaumatin‐like protein (TLP), a pathogenesis‐related (PR) protein, might be the candidate for the *Rj4* locus (Hayashi *et al*., 2014; Tang *et al*., 2016).

**Figure 3:**
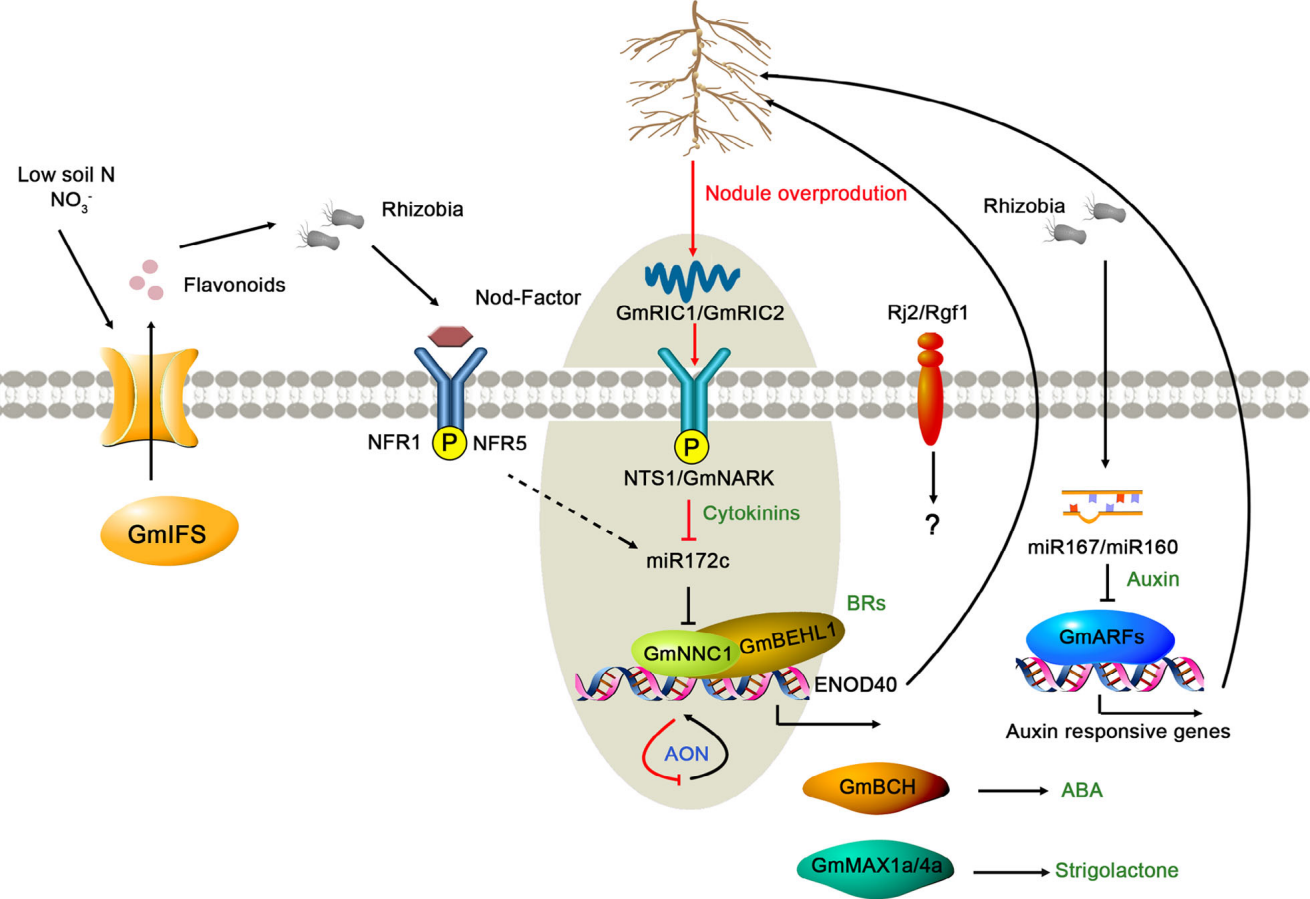
Proposed molecular network of soybean nodulation. Under low soil N, soybean plants produce flavonoids that trigger the production of bacterial Nod factors (NFR1 and NFR5), which, together with other signals, are perceived by receptors at the plasma membrane of plant epidermal cells. *Rj2*/*Rfg1* encodes a member of the Toll‐interleukin receptor/nucleotide‐binding site/leucine‐rich repeat (TIR‐NBS‐LRR) class of plant resistance (R) proteins that functions in symbiotic root nodules. Nodule overproduction is prevented by AON signalling, in which *GmRIC1* and *GmRIC2* activate GmNARK. GmNARK induces shoot‐derived cytokinins that, in turn, repress the transcriptional activity of miR172c. miR172c is a key positive regulator of nodulation and promotes the cleavage of the mRNA encoding its target gene *Nodule Number Control 1* (*NNC1*), which directly suppresses the transcription of the early nodulin gene *ENOD40*. Auxin, brassinosteroids (BRs), strigolactones, cytokinins and abscisic acid (ABA) are reported to function in nodulation in soybean. GmBEHL1, related to the BR signal, is an NNC1‐interacting protein. GmBEHL1 is suggested to be a co‐repressor of NNC1 and to negatively regulate soybean nodulation.

It has been suggested that flavonoids act as chemotactic signals to rhizobia under low‐N conditions in legumes (Liu and Murray, 2016; Subramanian *et al*., 2006). *GmMaT*, a malonyl‐CoA:flavonoid acyltransferase‐encoding gene, was found to catalyse isoflavone malonylation and affect malonyl isoflavone secretion, which had an effect on soybean nodulation (Ahmad *et al*., 2021). Flavonoids and/or related compounds such as isoflavones are released from legume roots and attract the Nod factors (NFs), which are secreted by rhizobia to initiate symbiotic nitrogen fixation (Figure 3). Soybean isoflavone synthase (IFS), a key enzyme in the biosynthesis of isoflavones, was induced by *Bradyrhizobium japonicum*. When the expression of *IFS* was knock down in soybean hairy root composite plants, these plants showed severely reduced nodulation (Liu and Murray, 2016; Subramanian *et al*., 2004; Subramanian *et al*., 2006). Additionally, glycolysis and lipid biosynthesis may also play essential roles in nodule formation (Chen *et al*., 2020a; Gillman *et al*., 2014; Lakhssassi *et al*., 2020; Zhang *et al*., 2020b).

The number of nodules formed on the roots of soybean is systemically controlled by autoregulation of nodulation (AON) (Caetano‐Anolles and Gresshoff, 1991). NTS1/GmNARK was found to be an important sensor that controls nodulation (Searle *et al*., 2003). Mutation of the *NTS1/GmNARK* locus led to prolific nodulation (Carroll *et al*., 1985a; Searle *et al*., 2003). Another two signalling molecules, *Bradyrhizobium‐induced and acting systemically 1* (*GmRIC1*) and *GmRIC2*, were involved in the transition of long‐distance signals from root to shoot. Further investigation showed that *GmNARK* can be activated by *GmRIC1* and *GmRIC2* (Reid *et al*., 2012). Overexpression of Gm*RIC1* and Gm*RIC2* strongly suppressed the nodulation in a *GmNARK‐*dependent manner (Reid *et al*., 2011) (Figure 3). An inoculation‐ and NARK‐dependent gene (*GmUFD1a*) responds in both the bioassay and intact, inoculated plants, indicating that it might be a novel component of the autoregulation pathway (Reid *et al*., 2012). The transcription factors GmNF‐YA1a and b were also identified as positive regulators in AON (Schaarschmidt *et al*., 2013). Overall, *GmNIC1* (nitrate‐induced and acting locally) and *GmRIC1* played key roles in AON and were relied on the activity of the nodulation autoregulation receptor kinase GmNARK (Reid *et al*., 2013).

Nitrogen and nitrogen compounds such as nitrate have been reported to negatively control nodulation (Carroll *et al*., 1985a,b; Day *et al*., 2010; Lim *et al*., 2014; Reid *et al*., 2011; Tanaka *et al*., 1985). The processes of nitrogen regulation of nodulation are distinct from AON and act immediately. Interestingly, a leucine‐rich receptor‐like kinase, *GmNARK*, was shared between AON and nitrate‐dependent regulation of nodulation (Ferguson *et al*., 2019). Under high nitrate, loss‐of‐function *GmNARK* mutants, *nts* (*nitrate tolerant symbiosis*), still exhibited super nodulation (Carroll *et al*., 1985b). GmNARK perceived the nodulation‐suppressing CLE peptides, such as GmRIC1 and GmRIC2, in the shoot through the AON pathway and sensed the nodulation‐suppressing CLE peptides, such as GmNIC1, in the root through nitrogen regulation of nodulation (Ferguson *et al*., 2019; Lim *et al*., 2014; Reid *et al*., 2011).

The development of nodules is significantly affected by phosphate (Pi). Pi starvation severely inhibited both nodulation and biological N_2_ fixation (Hernandez *et al*., 2009), with decreased nodule numbers, nodule size and nitrogenase activity of soybean. Two phosphate transporters, *GmPT5* and *GmPT7*, regulated phosphate transport and in turn affected nodulation in soybean (Chen *et al*., 2019; Qin *et al*., 2012). *GmPT5* controlled Pi entry from roots to nodules, was critical for maintaining Pi homeostasis in nodules and subsequently regulated nodulation and growth performance (Qin *et al*., 2012). Overexpression of *GmPT7* promoted nodulation and increased plant biomass, shoot nitrogen and phosphorus contents, improving soybean yield by up to 36% (Chen *et al*., 2019). The proteins phosphate‐transporter 1 (PHT1) and its regulator phosphate–starvation–response 1 (PHR1) worked as a PHR1‐PHT1 module to maintain Pi homeostasis and affected nodule development (Lu *et al*., 2020a).

Hormones have long been known to control nodule organogenesis (Grunewald *et al*., 2009). The transcription factors BRI1‐EMS suppressor 1 (BES1)/brassinazole‐resistant 1 (BZR1) played key roles in the brassinosteroids (BRs) signalling pathway (Yan *et al*., 2018). *GmBEHL1* was identified as an *Arabidopsis BES1/BZR1* homolog and can interact with NODULE NUMBER CONTROL 1 (NNC1), a transcriptional repressor that mediates soybean nodulation (Wang *et al*., 2014c; Yan *et al*., 2018). Knockdown and overexpression of *GmBEHL1* increased and decreased the number of nodules, respectively (Yan *et al*., 2018). The strigolactone (SL) biosynthesis enzymes *GmMAX1a* and *GmMAX4a* were apparently regulated by rhizobia infection. *GmMAX1a* and *GmMAX4a* knockdown lines exhibited decreased nodule number (Rehman *et al*., 2018) (Figure 3). GmMAX2 interacted with D14 and KAI to influence the SL and karrikins (KARs) signalling pathways to affect soybean root nodulation (Ahmad *et al*., 2020). Among the *YUCCA* (*YUC*) gene family, *GmYUC2a* functioned as an important regulator of auxin biosynthesis to modulate nodulation (Wang *et al*., 2019f). The β‐carotene hydroxylase GmBCH catalysed the conversion of β‐carotene to β‐zeaxanthin, which was related to the ABA synthesis pathway. RNAi‐mediated silencing of *GmBCH1/2* impaired nodule development and symbiotic nitrogen fixation (Kim *et al*., 2013).

It was also found that quite a few miRNA families showed transcript‐level responses to nodulation (Jin *et al*., 2018; Yan *et al*., 2015; Yan *et al*., 2016). For example, *miR393j‐3p* was significantly up‐regulated during nodule formation, and ectopic expression of *miR393j‐3p* significantly reduced nodule formation (Yan *et al*., 2015). The function of miR393 may be through regulation of GmTIR1/GmAFB3‐based auxin perception (Cai *et al*., 2017). miR172 is another important miRNA that was essential for nodule development. Overexpression of *MIR172* or the miRNA‐encoded peptide miPEP172c both resulted in an increase in nodule numbers in transgenic soybean roots (Couzigou *et al*., 2016; Yan *et al*., 2013). Further investigation showed that the function of miR172c was through the GmNINa‐miR172c‐NNC1 regulatory module (Wang *et al*., 2019c; Wang *et al*., 2014c) (Figure 3). *MIR160* promoted auxin activity by suppressing the levels of the ARF10/16/17 transcription factors to direct proper nodule formation and maturation in soybean (Nizampatnam *et al*., 2015; Turner *et al*., 2013). The expression levels of miR167 and its target were up‐ and down‐regulated by auxin in soybean. Moreover, miR167 could positively regulate nodule numbers by repressing the target genes *GmARF8a* and *GmARF8b*, which were homologous genes of the *Arabidopsis* auxin response factor *AtARF8* (Wang *et al*., 2015c) (Figure 3). In addition, mis‐expression of miR482, miR1512 and miR1515 increased nodulation (Li *et al*., 2010). Recently, Ren *et al*. showed that small RNA fragments (tRFs) derived from rhizobial transfer RNA (tRNA) serve as signal molecules that regulate host nodulation. Three families of rhizobial tRFs (Bj‐tRF001, Bj‐tRF002 and Bj‐tRF003) were confirmed to regulate host genes associated with nodule initiation and development (Ren *et al*., 2019), which represented a new evidence of a root‐shoot‐root signalling mechanism during nodulation (Shine *et al*., 2019; Zhang *et al*., 2020a).

The soybean gene *early nodulin 40* (*ENOD40*) played a pivotal role in nodule organogenesis (Charon *et al*., 1999; Kumagai *et al*., 2006; Wan *et al*., 2007). NNC1 regulated the expression of *ENOD40* by binding to the AP2 cis‐elements of *ENOD40* promoter, which consequently represses *ENOD40* expression and negatively regulated nodulation (Wang *et al*., 2014c). Several other genes that could affect soybean nodulation were also identified, such as *FW2.2‐like 1* (*GmFWL1*) (Libault *et al*., 2010), LysM‐type receptor kinase (*GmNFR1alpha*) (Indrasumunar *et al*., 2011), ecto‐apyrase (*GS52*) (Tanaka *et al*., 2011), ureide transporter (*UPS1*) (Collier and Tegeder, 2012), *symbiotic ammonium transporter 1* (*SAT1*) (Chiasson *et al*., 2014), *GmEXPB2* (Li *et al*., 2015a) and *VAMP721a* and *VAMP721d* (Gavrin *et al*., 2016). The transcription of the gene *target of rapamycin* (*GmTOR*) and its key downstream effector, *GmS6K1*, were activated during nodulation. When *GmS6K1* was knocked down, nodule development was severely impaired, suggesting an important role for the rapamycin pathway in nodule development (Um *et al*., 2013). *GmVTL1a*, which function as a transporter of ferrous iron from the infected root cell cytosol to the symbiosome, moved iron across the symbiosome membrane to bacteria’s and played a crucial role in nitrogen fixation (Brear *et al*., 2020; Liu *et al*., 2020d). In addition, nodulation was affected under acidic and drought conditions (Gil‐Quintana *et al*., 2013; Lin *et al*., 2012). A very recent study reported that *Nodule Number Locus 1* (*GmNNL1*), which encodes a novel R protein, may trigger immunity and inhibit nodulation (Zhang *et al*., 2021).

### Domestication

Plant domestication is one of the most important aspects contributing to the development of agriculture (Diamond, 2002). In addition to investigations at the population level through the resequencing of germplasm (Lam *et al*., 2010; Li *et al*., 2013b; Sedivy *et al*., 2017; Zhou *et al*., 2015), some genes responsible for traits linked to soybean domestication were identified.

One important agronomic trait that was targeted by human selection during crop domestication is decreased pod shattering and seed dispersal (Sedivy *et al*., 2017). *SHAT1‐5*, a gene encoding a NAC (NAM, ATAF1/2 and CUC2) transcription factor, was found to be a prime domestication gene, and the allele in cultivated soybean improves the thickening of the fibre cap cells and suppresses pod shattering (Dong *et al*., 2014). *Pdh1* is another gene that affects the pod shattering phenotype and showed artificial selection in landraces of Japan, Korea and other South‐East Asian countries. The cultivated alleles promoted torsion of dried pods under low humidity, causing higher pod dehiscence (Funatsuki *et al*., 2014).

Loss of dormancy is another important domestication trait and relates to both physiology and physical, structural changes (Finch‐Savage and Leubner‐Metzger, 2006). In soybean, *Hs1‐1* has long been identified as an important locus corresponding to loss of dormancy through a physical change (Liu *et al*., 2007). Sun *et al*. performed a genetic analysis and suggested that *Hs1‐1* encodes a calcineurin‐like metallophosphoesterase transmembrane protein (Sun *et al*., 2015a), while Jang *et al*. reported that a single nucleotide polymorphism in another gene, a endo‐1,4‐β‐glucanase, in this region may be responsible for *Hs1‐1* (Jang *et al*., 2015). Another key locus responsible for seed dormancy is *G*, which was found to encode a CAAX amino‐terminal protease protein. Interestingly, *G* had undergone parallel selection across different plant families (Wang *et al*., 2018b), which may make it a candidate gene for the acceleration of crop improvement (Lyu, 2018; Rendon‐Anaya and Herrera‐Estrella, 2018; Wei and Huang, 2018).

As a short‐day flowering plant, the spread of soybean cultivation latitudinally requires adaptation to new photoperiods. Several flowering‐related genes in soybean have been selected during human cultivation (Cober *et al*., 2010; Kim *et al*., 2012a). *GmCRY1a* and *GmCOL7a*, soybean homologs of *Arabidopsis CRYPTOCHROME 2* (*CRY2*) and *CONSTANS* (*CO*), respectively, were reported to exhibit strong selection signatures (Li *et al*., 2013b; Wang *et al*., 2016c; Wu *et al*., 2014; Zhang *et al*., 2008). The major maturity loci *E1*, *E2*, *E3* and *E4*, where several flowering‐associated genes are located, have contributed to local adaptation (Kanazawa *et al*., 2009; Liu *et al*., 2008; Wang *et al*., 2016c; Watanabe *et al*., 2009; Watanabe *et al*., 2011; Xia *et al*., 2012; Zhai *et al*., 2014b). A recent study found that homologous *pseudo‐response‐regulator* (*PRR*), *Tof11* and *Tof12*, had undergone strong selection (Lu *et al*., 2020b). Several yield‐related genes, such as *GA20OX*, *NFYA* (Lu *et al*., 2016), *Dt1* (Tian *et al*., 2010), *SoyWRKY15a* (Gu *et al*., 2017) and *PP2C‐1* (Lu *et al*., 2017b), had also undergone selection during soybean domestication.

## Transformation technology

### Transgenic technology

Soybean transgenic technology is a necessary tool for soybean gene function study. Genetic transformation of soybean has been studied for over two decades; however, the progress remains slow and inefficient, which is why the functional validation in some studies was performed in *Arabidopsis* instead of soybean. Several transformation systems were developed, including using shoot meristems (Mccabe *et al*., 1988; Rech *et al*., 2008), hypocotyls (Aragão *et al*., 2000; Dan and Reichert, 1999; Wang and Xu, 2008), embryo (Finer and Mcmullen, 1991; Trick and Finer, 1998), immature cotyledons, half‐seed explants (Liu *et al*., 2004; Paz *et al*., 2006) and cotyledonary nodes (Li *et al*., 2017c; Liu *et al*., 2004; Sato *et al*., 1993). Considering the operation, reproducibility, copy number of foreign DNA and experimental cost, *Agrobacterium*‐mediated cotyledonary node (CN) soybean transformation was commonly used nowadays (Hinchee *et al*., 1988; Paz *et al*., 2006; Somers *et al*., 2003). The overall average transformation efficiency was 3.8%–8.7% (Li *et al*., 2017c; Paz *et al*., 2006). Recently, Pareddy *et al*. (2020) enhanced the average transformation efficiency to 18.7%. But it is still lower than that in rice of 23% (Ge *et al*., 2006; Lin and Zhang, 2005) and maize of over 30% (Ishida *et al*., 1996; Yang *et al*., 2006).

**Figure 4:**
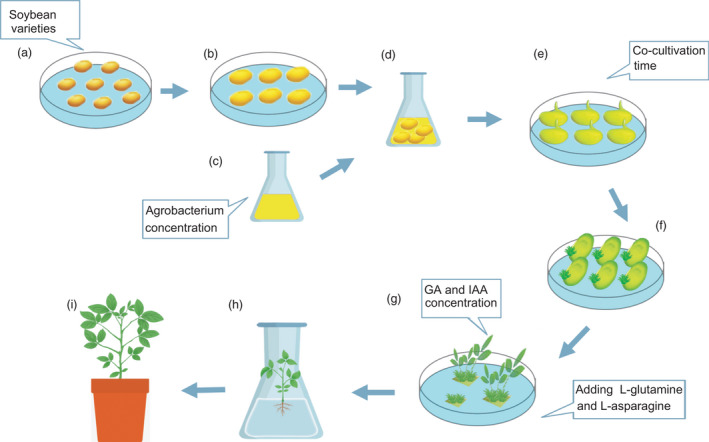
General procedure of *Agrobacterium*‐mediated cotyledonary node soybean transformation. (a) Seed sterilization. Selection of soybean varieties dominates the efficiency of transformation affecting on Agrobacterium infection and regeneration capacity (Song *et al*., 2013c). (b) Seed imbibition. (c) Preparation of *Agrobacterium*. (d) *Agrobacterium* infection. It is one of the most important steps, *Agrobacterium* concentrations, soybean explants, *Agrobacterium* suspension medium, and co‐cultivation time will affect the infection efficiency. (e) Co‐cultivation. Suitable concentration of plant hormone is necessary to (f) Shoot induction and (g) shoot elongation that will improve the efficiency (Li *et al*., 2017c). Adding L‐glutamine and L‐asparagine together into culture media will increase the transformation frequency of soybean by suppressed the expression level of *GmPRs* (Chen *et al*., 2018a). (h) Rooting. (i) Transplanting. Words in the blue boxes indicate the methods of improving infection efficiency.

The general transformation process includes seed sterilization and germination; *Agrobacterium* infection; co‐cultivate soybean explants and *Agrobacterium*; shoot induction; shoot elongation; rooting; and at last transferring the plants to pots containing soil (Figure 4). In these processes, many factors can affect the efficiency. The first effector is soybean genotypes. Song *et al*. compared transformation efficiency and regeneration rate of twenty soybean varieties and found that transformation efficiency between different varieties differed significantly (0.31%–4.59%) (Song *et al*., 2013c). Secondly, in the process of *Agrobacterium* infection which is one of the most important steps, all of *Agrobacterium* concentrations, soybean explant status, *Agrobacterium* suspension medium and co‐cultivation time will affect the infection efficiency. Another key process in determining the transformation efficiency is explant regeneration. It was reported that plant hormone plays critical role in inducing the regeneration of explant and its suitable concentration could improve the efficiency (Li *et al*., 2017c). Chen *et al*. (2018a) reported that adding L‐glutamine and L‐asparagine together into culture media will increase the transformation efficiency by suppressing the expression of *GmPRs*.

### Genome editing

Genome editing can introduce precise modifications into the genome to obtain predictable and desired traits, which has been proved to be a powerful approach for functional study and molecular design breeding than the traditional genetics approaches, such as mutagenesis, transgenic RNAi or overexpression (Gao, 2021; Rodriguez‐Leal *et al*., 2017). Of the different genome editing systems, CRISPR (clustered regularly interspaced short palindromic repeat)/Cas (CRISPR‐associated) system shows high efficiency and has been extensive applied in different species (Feng *et al*., 2013; Jiang *et al*., 2013; Miao *et al*., 2013; Shan *et al*., 2013; Svitashev *et al*., 2015; Upadhyay *et al*., 2013).

The first knock out and DNA homology‐directed recombination (HDR) soybean plant created by CRISPR/Cas9 technology was successfully obtained in 2015 (Li *et al*., 2015b). In 2016, Du *et al*. (2016) found that changing the *AtU6‐26* promoter into *GmU6‐16g‐1* promoter of the CRISPR/Cas9 system could significantly improve the efficiency of targeted mutagenesis in soybean. In soybean, nearly 75% of the genes present in multiple copies and knockout of a single gene usually does not exhibit mutant phenotype. It is important to develop a dedicated CRISPR/Cas9 system that can edit multiple homologous genes simultaneously. By optimizing the steps of vector construction, sgRNA assessment, pooled transformation and sgRNA identification, a CRISPR/Cas9 system that can generate multiplex mutagenesis with higher efficiency was developed (Bai *et al*., 2020). In nature, beside the alleles caused by loss‐of‐function mutations, large part of the phenotypic variations in agronomic traits are resulted from single nucleotide polymorphism (SNP) variations. Damage of the function of the whole gene using gene‐editing system usually leads to severe phenotype, which may not be optimizable for agronomic trait improvement in production. Therefore, generation of point mutations at specific sites affecting important agronomic traits is of great value in molecular breeding (Mishra *et al*., 2020). Recently, ‘base editing’ has been developed from CRISPR/Cas9 system, which converts single base into another without requiring DNA double‐strand breaks or a donor template (Komor *et al*., 2016). Cai *et al*. successful applied the system to create point mutant of *GmFT2a* and *GmFT4* (Cai *et al*., 2020a).

Nowadays, CRISPR/Cas9 was widely applied in soybean functional studies (Cai *et al*., 2018; Du *et al*., 2016; Li *et al*., 2019a; Michno *et al*., 2015; Sun *et al*., 2015b; Xu *et al*., 2020). For instance in identification of the genes controlling flowering time, frameshift mutations generated by CRISPR/Cas9 demonstrated that *GmFT2a* mainly function under short day (SD), whereas *GmFT5a* had more significant effects under long day (LD) (Cai *et al*., 2018; Cai *et al*., 2020b). Similarly, knockout of *GmPRR37* by the CRISPR system suggested that it can repress flowering under LD (Wang *et al*., 2020c). Two CRISPR/Cas9 gene‐editing mutants of *Glyma.13G114200* exhibited male sterility phenotype, confirming that it was the casual gene for *GmMS1* for male sterility (Fang *et al*., 2021b; Jiang *et al*., 2021; Nadeem *et al*., 2021). CRISPR/Cas9 was also be applied in yield and seed quality‐related trait modifications, such as to alter plant architecture by editing *GmLHYs* (Cheng *et al*., 2019) or *SPL9* (Bao *et al*., 2019; Cai *et al*., 2018; Cheng *et al*., 2019), to increase seed number per pod by editing *Ln* (Cai *et al*., 2021), to reduce beany flavour by knocking out *LOXs* (Wang *et al*., 2020b), to increase isoflavone content by editing *GmF3H1*, *GmF3H2* and *GmFNSII‐1* simultaneously (Zhang *et al*., 2020f) and to alter the fatty acid profiling by editing *FAD2‐2* (Al Amin *et al*., 2019). In the future, more application of ‘base editing’ for single gene or for multiple genes simultaneously will greatly promote the functional study and molecular design breeding in soybean.

## Challenges and future perspectives

The Green Revolution is one of the most remarkable events in agriculture and greatly increased the production of major crops (Hedden, 2003). However, few improvements in yield have been made for soybean over the past six decades. In order to meet the needs of a growing world population, soybean yield must increase at a faster rate than it is at present (Ainsworth *et al*., 2012; Ray *et al*., 2013). There is an urgent need for a soybean ‘Green Revolution’ to breed supper varieties with the ideal plant architecture that are adapted to high‐density planting environment (Liu *et al*., 2020e). At this point, most soybean production occurs in South America, North America and Asia. In the future, Africa might become another dominant soybean production area. Therefore, there is a need to start studies on genetic improvement and production technologies for an Africa‐adapted soybean. Moreover, there is a need to develop new varieties to meet future environmental changes and to create a more sustainable agricultural system (Bishop *et al*., 2015; Kumagai *et al*., 2015; Mourtzinis *et al*., 2015; Ruiz‐Vera *et al*., 2013; Tian *et al*., 2020).

To create a super variety, breeders normally need to stack multiple desirable traits into a single line. However, most important traits are quantitatively controlled and exhibit inherited correlations. Bringing about a ‘Green Revolution’ in soybean may prove to be an exacting task because of the unique plant architecture and the complicated components that determine the final yield in soybean (Liu *et al*., 2020e). Understanding the inherited mechanism of each trait and the regulatory network among different traits will help us to design the desired crops (Tian *et al*., 2020). A dissection of the genetic networks underlying 84 agronomical traits has provided insights into the molecular design of soybean (Fang *et al*., 2017).

*De novo* domestication of new crops aims to rapidly domesticate wild or semi‐wild plants into agricultural crops with favoured agronomic traits by utilization of combines modern technologies, including genomics, gene editing and synthetic biology (Fernie and Yan, 2019; Khan *et al*., 2019; Li *et al*., 2018b; Yu *et al*., 2021; Zsogon *et al*., 2018). Wild soybean showed higher protein content, lower oil content and higher stress tolerance, whereas most of the current cultivated soybeans exhibited relatively lower protein content and higher oil content. So far, not many genes related to soybean domestication has been identified, leaving the domestication traits are still poorly understood. For instance, switching plant architecture from twinning (sprawl) habit in wild soybeans to upright habit in cultivated soybeans is one of the most prerequisite domestication trait for soybean; however, a genetics dissection of this important trait is unclear yet. With the rapid development of functional genomics in soybean, an ever‐increasing number of genes related to agronomic traits are being cloned, which may enable us to re‐domesticate the wild soybean into a new crop keeping the characters of higher protein content and higher stress tolerance.

At this time, soybean functional studies and breeding still face some technical challenges. The lack of a stable and high‐efficiency transgenic system is one of the biggest challenges, which makes functional study more time‐consuming than in species with well‐developed transformation systems, such as rice and *Arabidopsis*. Another challenge is phenotyping. Because soybean is quite sensitive to photoperiod, the phenotypes of a soybean line usually exhibit significant variations in different environments, which make precise phenotyping and functional study more difficult. The recent development of new technologies, such as target base editing (Cai *et al*., 2020a) and a transient expression system (Xiong *et al*., 2019), will facilitate soybean functional studies. Moreover, the recently developed graph‐based soybean pan‐genome will both rejuvenate previous omics data and revolutionize functional and evolutionary genomic studies in soybean (Liu *et al*., 2020f; Liu and Tian, 2020).

## Conflict interest

We declare that we have no conflict of interest.

## Author contributions

M.Z., S.L., Z.W., Y.Y., Z.Z., Q.L., X.Y., Z.D., F.K., B.L., B.R. and Z.T. drafted the manuscript. Z.T. conceived the article and revised the manuscript.

## Funding

This work was supported by the National Natural Science Foundation of China (31801380), the National Key Research & Development Program of China (2017YFD0101305), and the State Key Laboratory of Plant Cell and Chromosome Engineering (PCCE‐KF‐2019‐05).

## Supporting information


**Table S1** Representative genes related to agronomically important traits in soybean.
